# Time-elapsed microstructural imaging of failure of the reverse shoulder implant

**DOI:** 10.1186/s13018-024-04652-9

**Published:** 2024-03-12

**Authors:** Saulo Martelli, Egon Perilli, Xiaolong Fan, Sophie Rapagna, Ashish Gupta

**Affiliations:** 1 School of Mechanical Medical and Process Engineering, Queensland University of Technology, Gardens Point Campus, P’Block, Level 7, Room 717, Brisbane, QLD 4000 Australia; 2 Medical Devices Research Institute, College of Science and Engineering, Flinders University, Adelaide, SA Australia; 3 Greenslopes Private Hospital, Brisbane, QLD Australia; 4 Queensland Unit for Advanced Shoulder Research (QUASR), Queensland University of Technology, Brisbane, QLD Australia

**Keywords:** Reverse shoulder arthroplasty, Implant failure, Bone microstructural imaging, Biomechanics

## Abstract

**Background:**

Reverse Shoulder Arthroplasties (RSA) have become a primary choice for improving shoulder function and pain. However, the biomechanical failure mechanism of the humeral component is still unclear. The present study reports a novel protocol for microstructural imaging of the entire humerus implant under load before and after fracture.

**Methods:**

A humerus specimen was obtained from a 75-year-old male donor. An expert surgeon implanted the specimen with a commonly used RSA implant (Aequalis reversed II, Stryker Orthopaedics, USA) and surgical procedure. The physiological glenohumeral contact force that maximized the distal implant migration was selected from a public repository (orthoload.com). Imaging and concomitant mechanical testing were performed using a large-volume micro-CT scanner (Nikon XT H 225 ST) and a custom-made compressive stage. Both when intact and once implanted, the specimen was tested under a pre-load and by imposing a constant deformation causing a physiological reaction load (650 N, 10 degrees adducted). The deformation of the implanted specimen was then increased up to fracture, which was identified by a sudden drop of the reaction force, and the specimen was then re-scanned.

**Results:**

The specimen’s stiffness decreased from 874 N/mm to 464 N/mm after implantation, producing movements of the bone-implant interface consistent with the implant’s long-term stability reported in the literature. The micro-CT images displayed fracture of the tuberosity, caused by a combined compression and circumferential tension, induced by the distal migration of the implant.

**Conclusion:**

The developed protocol offers detailed information on implant mechanics under load relative to intact conditions and fracture, providing insights into the failure mechanics of RSA implants. This protocol can be used to inform future implant design and surgical technique improvements.

## Background

In the last 15 years, the number of Reverse Shoulder Arthroplasties (RSA) has increased exponentially as a choice for pain relief, improving shoulder function and implant survival [1]. Revision rates have been reported between 12% and 16% [1, 2], with exceptions reaching 69% [3]. Aseptic loosening of the humeral component, and intra- and post-operative bone fracture, are among the ten most common complications potentially leading to revision surgery [1]. In this context, large-volume micro-computed tomography (micro-CT) scanners enable visualization of the microstructure of entire bones [4–7], joints [8] and implants [7] under controlled loading, for advanced analyses of biomechanical failure of implantable devices.

The engineering quest for failure-free designs benefits from a systematic risk analysis of biomechanical failure linking the different, clinically observable scenarios of failure, the failure modes potentially leading to each scenario, the corresponding driving biomechanical parameters, and criteria for risk assessment [9, 10]. Aseptic loosening is the most complex scenario of failure to eventuate due to several concurrent failure modes, including damage of the peri-prosthetic bone, fibrotic bone tissue differentiation, and adverse bone resorption [10]. Overload most likely causes intra-operative bone fractures whereas, post-operatively, overload and cyclic damage accumulation, or fatigue, can cause bone and implant fracture [10]. Concerning the driving mechanical parameters and a criterion for risk assessment, no bone damage is expected for strain levels below yield (0.7–1.1%) [11]. A soft fibrotic tissue differentiation prevents effective osseointegration for movements of the bone-implant interface exceeding 0.15 mm, while an implant subsidence, generated by both elastic bone deformation and interfacial motion, equal to 1.2 mm was associated with a revision risk of more than 50% in hip implants [12, 13]. Adverse bone resorption via stress shielding occurs in bone regions experiencing a 70% post-operative reduction of strain energy density [14]. Cortical strain, fracture load, and stiffness are mechanical parameters related to fracture, which can be analyzed relative to pre-operative intact conditions to account for their large variability across people [15]. Finally, the risk analysis should consider, among implant loads caused by common daily activities in implanted patients, the loading conditions causing the highest risk of implant failure, to draw meaningful considerations of implant safety [10, 16–18]. Therefore, measuring the displacement of the bone in intact and implanted conditions under controlled load can enable a complete risk analysis of biomechanical failure.

A relatively small number of time-elapsed micro-CT studies of entire human bones and joints have focused on different anatomical regions and objectives [4]. The microstructural fracture mechanism has been studied on thoracic vertebral bodies and proximal femurs, showing synergic region- and load-specific cortical and trabecular interdependency in determining bone’s support capacity [4, 6, 19]. In operated joints, the relative movement of tibial fragments, surgically reduced using a CoCr locking plate and screws, was quantified under simulated gait loads [8], and a zero-strain error analysis in peri-prosthetic bone was performed for titanium tibial trays [7]. Micro-CT and digital volume correlation were demonstrated to provide strain measurements of the glenoid (MAER = 694 µe), enabling the comparison of different implant designs and surgical methods [20], reverse engineer bone material properties of both the glenoid and the proximal humeral head [21], and advance modeling technologies [5]. Yet, to the best of the authors’ knowledge, the volumetric displacements to failure of the entire humeral component of a reverse shoulder implant have not been reported.

The present study aimed to develop a novel protocol for the microstructural imaging of the entire proximal half of the humerus hosting a commonly used RSA implant, while loaded by imposing to the specimen three constant deformation states stepwise increased up to cause fracture of the specimen. The protocol was developed for a selected donor, the Aequalis reversed II stem (Stryker Orthopaedics, USA), and the inlay surgical procedure. The loading configuration that maximized the implant distal migration was obtained from a public repository of glenohumeral contact forces in implanted patients, for various types of normal activity [22]. The micro-CT imaging and testing methods were adapted from earlier experiments focusing on the femur and the tibia [7, 19]. The intact humerus was imaged first under minimal load (pre-load) and then non-destructively under the selected loading condition. After implantation, the non-destructive testing and imaging were repeated for the implanted humerus. The displacement was then further increased until causing a sudden drop of the measured compression force, and the specimen was re-imaged. The reaction force was continuously measured. The displacement of the construct boundaries, implant and bone microstructure were obtained from the micro-CT images and compared.

## Methods

### The specimen

The left humerus from a male donor who was 75 years of age at death, presenting no signs of skeletal deformity or abnormality, was obtained through the institutional Medical Engineering Research Facility (MERF) of the Queensland University of Technology. Soft tissues were removed using a scalpel. The humerus was wrapped in a cloth soaked with phosphate-buffered saline (PBS) solution and stored in a freezer at − 20 °C. Ethics clearance was obtained from the institutional Research Ethics Committee (ethics # 2,021,000,088).

### The loading configuration

The glenohumeral contact force in five patients wearing a telemetric anatomical shoulder replacement executing 16 different activities was downloaded from a publicly available database (https://orthoload.com) [22], overall providing 56 tasks of motion and over 83,000 data points. The direction of the force, selected for the experiment, was the one most aligned with the diaphyseal axis, a condition expected to maximize the distal migration of the implant per unit of load applied. Forces below 400 N were excluded from the analysis.

### Specimen preparation

**Fig. 1:**
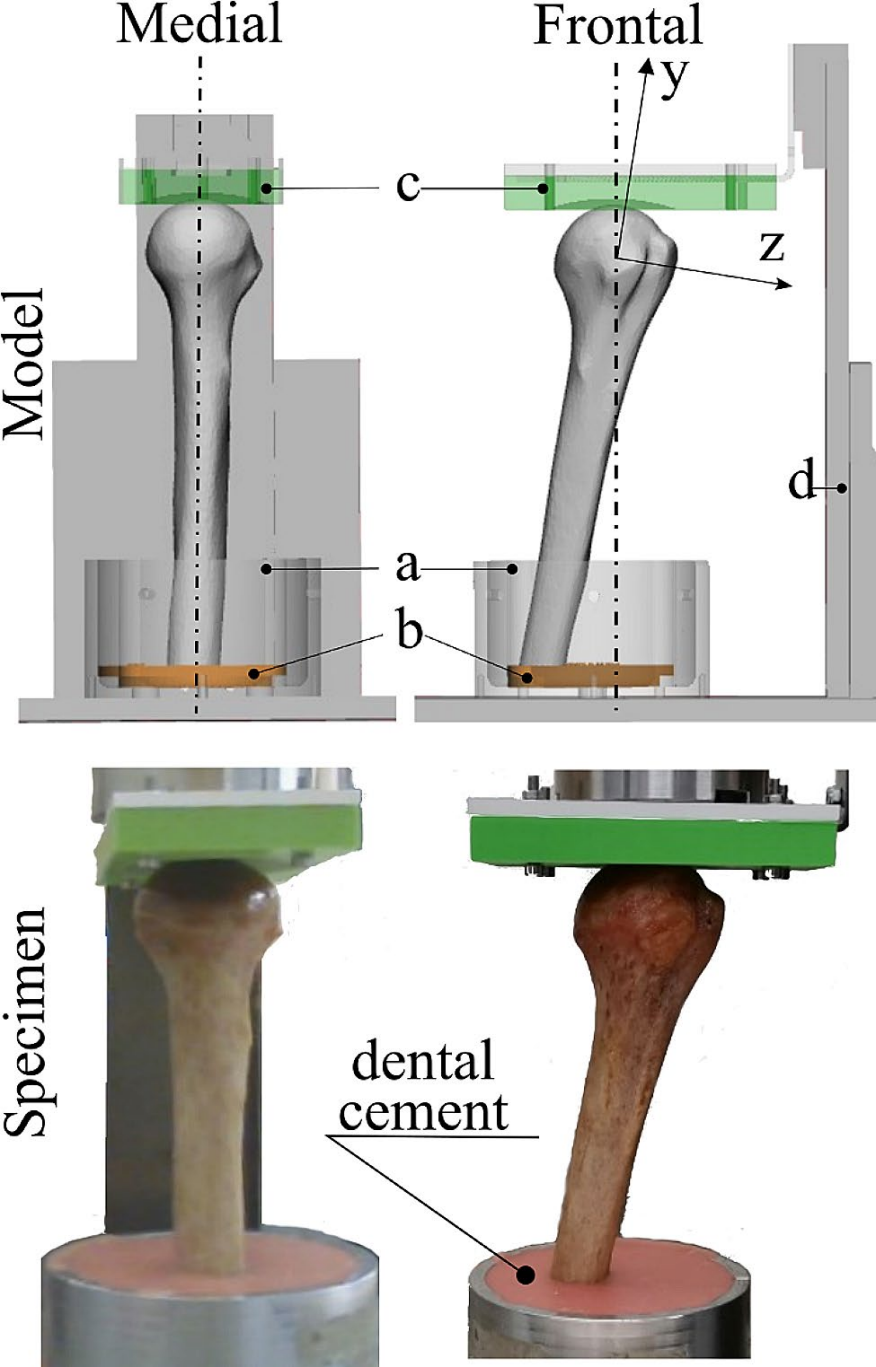
Top row: the medial and frontal views of the 3D model of the specimen aligned to the cup in the alignment rig, displaying the shaded aluminum cup (**a**), the 3D printed holder of the distal humerus (**b**), and the spherically shaped concave 3D printed holder of the head of the specimen (in green color) (**c**). Bottom row: photos of the specimen after potting, in corresponding medial and frontal views

The specimen was thawed for 24 h and scanned using a clinical CT scanner (Aquilion 54, Toshiba Medical Systems) using a helical scanning protocol (voltage: 120 kV; tube current: 300 mA; 0.32 mm slice thickness; 0.605 mm pixels size). The bone geometry was segmented from the images using a semi-automatic threshold-based procedure implemented in Mimics (Materialize NV, Leuven, Belgium). The diaphysis was cut at 220 mm from the proximal end of the humerus head. The specimen was aligned to the aluminum cup using a custom alignment rig and later mounted on the compressive stage. The humerus head was aligned to the vertical axis of the cup, while the diaphysis was abducted 10° in the frontal plane. A three-dimensionally (3D) printed insert was created for holding in place the distal diaphysis of the specimen while potting (3-Matic, Materialize NV, Leuven, Belgium), using a Lulzbot Mini 2 3D printer with ASA (acrylonitrile styrene acrylate). The specimen was potted distally 55 mm deep using dental cement (Soesterberg, The Netherlands), which met the ISO 5833 requirements (Fig. 1).

**Fig. 2:**
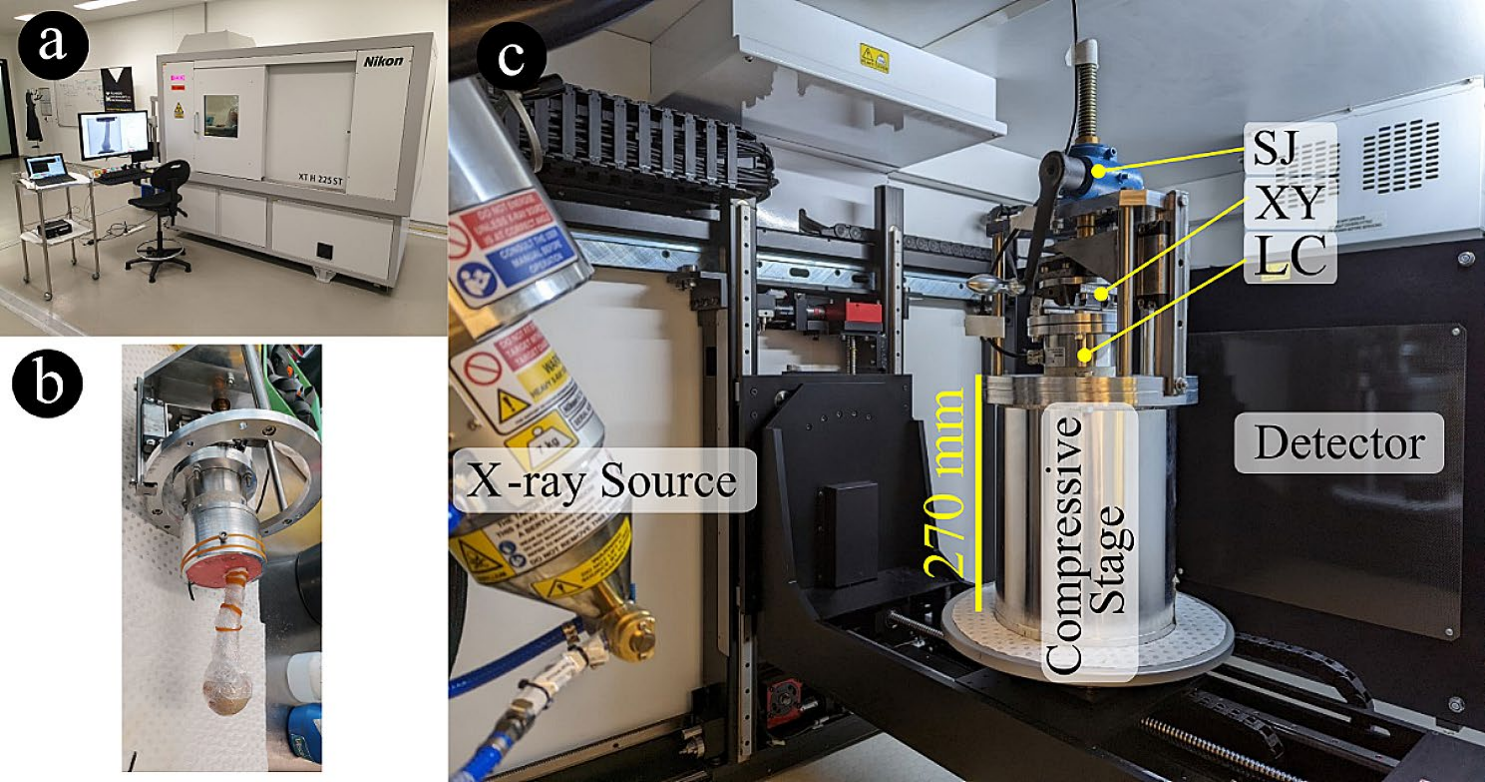
Microstructural imaging of the humerus under load. From the left-hand side, (**a**) the micro-CT scanner, with large computer screen displaying radiographic projections during scanning, and laptop screen monitoring the compressive force applied to the specimen; (**b**) the specimen (potted in dental cement and wrapped in cling wrap to maintain bone moisture throughout the experiment) ready to be mounted onto the compressive stage, and (**c**) inside of the micro-CT gantry, showing the compressive stage (containing the specimen) with the load cell, LC, the low friction table, XY, and the crank actuating the screw jack, SJ

The specimen and potting cup assembly were then mounted on the radio-transparent compressive stage described earlier [19]. In summary, the compressive stage comprised (1) a cylindrical 2 mm thick radio-transparent aluminum compression chamber, (2) a self-locking screw-jack mechanism (Benzlers, Örebro, Sweden, maximal load: 10,000 N gear ratio 27:1, 0.148 mm per revolution) controlling the vertical displacement of the specimen assembly, (3) a low-friction x-y table (THK Co., Tokyo, Japan) minimizing the transversal force components and, (4) a 6-degree-of-freedom load cell (ME-measurement systems GmbH, Hennigsdorf, GE; capacity: 10,000 N and 500 Nm; maximal error: 0.005%) (Fig. 2).

### Time-elapsed micro-CT imaging of the intact humerus

Micro-CT imaging was performed with a large-volume micro-CT system based at Flinders University (Nikon XT H 225 ST, Nikon Metrology, Tring, Hertfordshire, UK; 4056 × 4056 pixel array detector) (Fig. 2). The scanning settings were defined according to earlier guidelines [7]. The 4056 × 4056 pixels projection images provided a field of view equal to 182.5 × 182.5 mm (width × height) containing the entire specimen, including a portion of proximal and distal constraints (i.e., the pressure socket and the aluminum cup) at a 45 μm isotropic pixel size. The volume of images was acquired at a peak voltage of 215 kVp, current 209 µA (45 W), using a 0.1 mm tin filter, using a 0.21° rotation step over 360°, with 2 s exposure time and 1 × binning [7]. The total acquisition time was 57 min per scan. The intact specimen was scanned first under a 100 N pre-load, and then after a nominal 650 N compression was applied to the specimen. The initial position was defined by actuating the screw mechanism up to inducing a compressive reaction force equal to the pre-load. The nominal load increment was applied by actuating the screw-jack mechanism via a handle at a constant loading rate (0.5–0.75 revolutions per second, 0.148 mm/revolution, resulting in 0.074–0.111 mm/s). Specimen relaxation was allowed for no less than 10 min after each repeated scan. The 6-component reaction force was recorded throughout the experiment with a laptop computer [19]. At all times, the specimen was kept wrapped in saline-soaked paper surrounded by cling wrap to maintain bone moisture.

### Humerus implantation

**Fig. 3:**
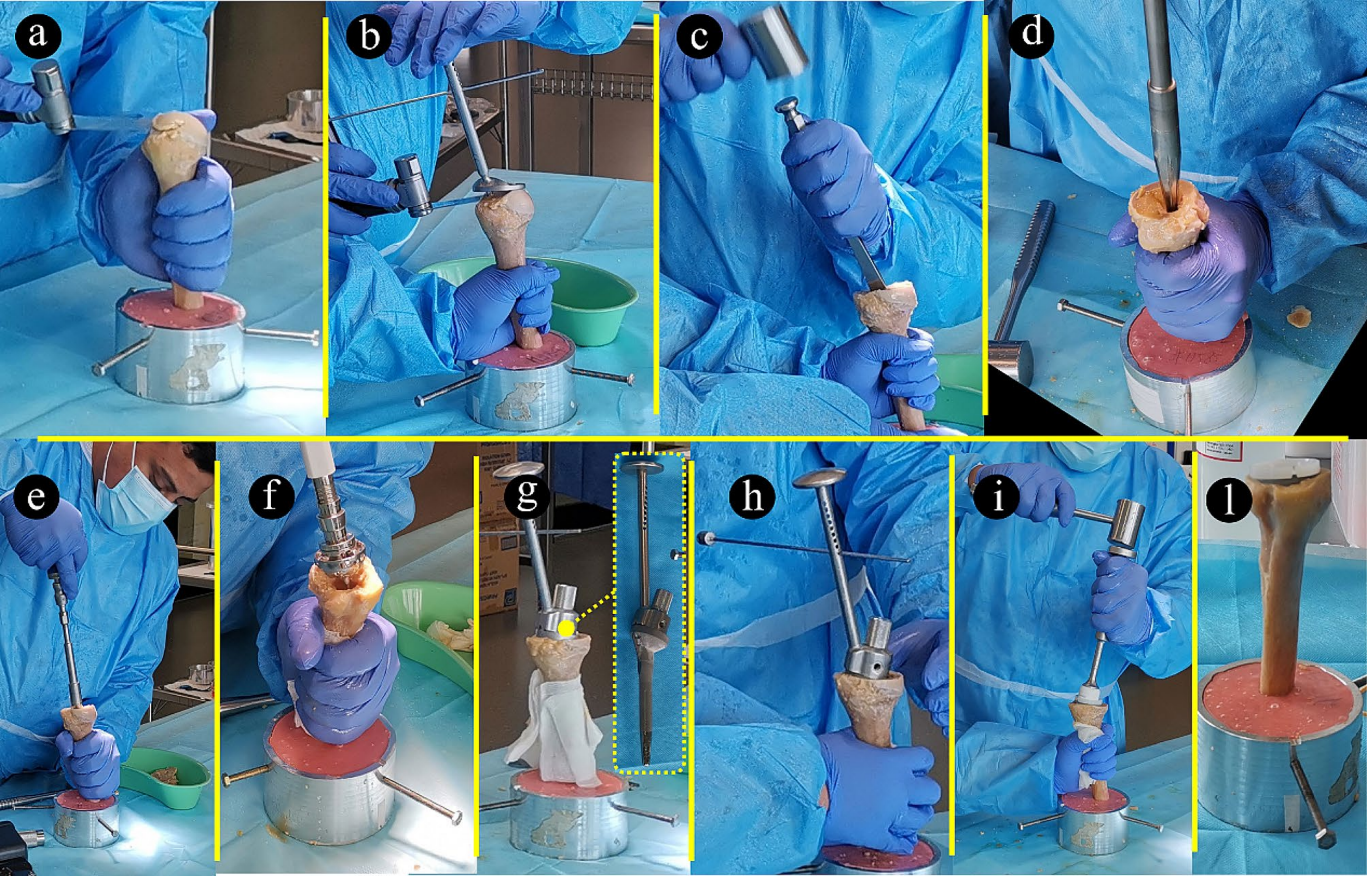
The implantation process: removal of cortical bone at humeral entry point (**a**); humeral head resection using cutting guide and version rod (**b**); metaphyseal bone harvest with osteotome (**c**); humeral reaming (**d**); progressive reaming technique for diaphyseal size (**e**); metaphyseal spherical reamer to match metaphyseal cup (**f**); humeral prosthesis insertion with impactor handle (**g**); prosthesis positioning using version rod (**h**); impaction of polyethylene insert using impactor (**i**); final construct showing inlay placement of prosthesis (**l**)

The humerus was implanted using an Aequalis reversed II implant (Stryker Inc.), using a standardized prescribed inlay technique (Fig. 3). The humeral implant component included a distal titanium stem (100 mm long, 15 mm diameter proximally, 6 mm diameter distally), a CoCr proximal metaphysis (36 mm maximum external diameter, 3–9 mm wall thickness), and a polyethylene insert. The humeral head was cut using an intramedullary resection guide at 155 degrees. The canal of the humerus was broached with the starting broach, and incremental diaphyseal broaching was carried out to ensure diaphyseal fit. The proximal epiphyseal reaming was carried out using motorized reamers. The implant was then assembled on the back table and press fitted for an inlay construct with a size-matched diaphyseal fitting component. The polyethylene component was then impacted as per the surgical technique recommended by the manufacturer.

### Time-elapsed micro-CT imaging of the implanted humerus

The implanted specimen was micro-CT imaged using the same protocol used for the intact humerus. The specimen in the initial position (under pre-load) was scanned. Then, the screw mechanism was manually actuated, at a constant speed, by applying an increase of the compressive force of 650 N to the specimen and re-imaged. Finally, the load was further increased until the load cell recorded a sudden drop in the compression force, denoting failure. The failed bone-implant construct was then micro-CT scanned by keeping the actuator of the compressive stage in a constant position.

### Data analysis

Axial micro-CT images were reconstructed using CTPro3D software (Nikon Metrology) and saved as 8-bit bitmap images (256 grey levels). For each scan, a stack of up to 3956 consecutive cross-sections was reconstructed, each 2558 × 2502 in size (115 × 112.6 mm) centered on the specimen, resulting in a height up to 178 mm, saved in 8-bit greyscale format, generating a dataset occupying 23.5 Gbyte.

The range of forces in the database was analyzed in terms of force magnitude or direction with respect to the humerus to provide context to the selected loading condition.

Reaction forces and moments were analyzed, plotted, and related to the corresponding displacement of the aluminum potting cup measured from the images. On the opposite side, the pressure socket was immobile during all scans. The specimen’s stiffness was calculated for the physiological load step using the increment in vertical displacement of the aluminum cup, and the increment in compression force recorded, relative to the pre-load condition.

The deformation of the humeral head, independent from that of the diaphysis, was analyzed by co-registering the micro-CT cross-section images at the humerus metaphysis, just below the humeral head. Bone-implant movements were analyzed by co-registering the micro-CT cross-section images, using as the registration target the portion of the images distal to the implant, including the cement and the rim of the aluminum cup. Co-registration was performed using Dataviewer (Skyscan–Bruker, Kontich, Belgium). The deformation of the bone, intact and implanted, under prescribed loadings, was analyzed using 2D and 3D micro-CT visual representations in Matlab (The MathWorks Inc., Natick, USA) and Drishti [23].

## Results

**Fig. 4:**
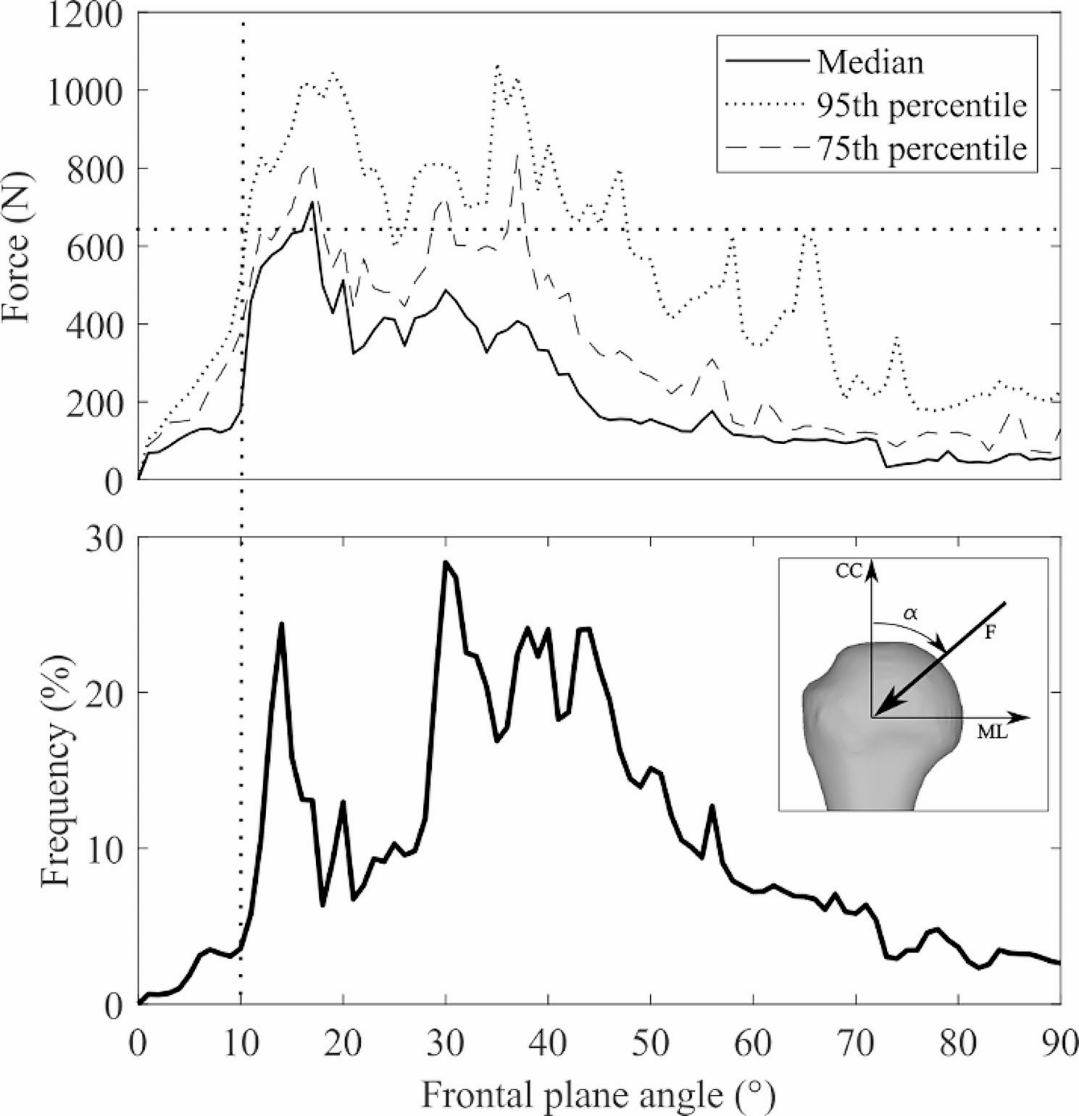
The magnitude (median, 74th, and 95th percentiles) of the glenohumeral contact force recorded in participants against the rotation angle in the frontal plane (top). The frequency panel (bottom) represents the number of forces recorded at each angle relative to the size of the dataset. The vertical and horizontal dash lines represent the experimental load direction and magnitude

The glenohumeral contact forces in the public database (orthoload.com) [22] peaked at 1761 N during the elevation of the arm using a 2 kg weight. The force direction spanned more than 90° rotation in the frontal plane. The force magnitude and frequency were maximal between 10º – 20º and 30º – 50º rotation. The peak median force was 713 N at 15º rotation (Fig. 4).

**Fig. 5:**
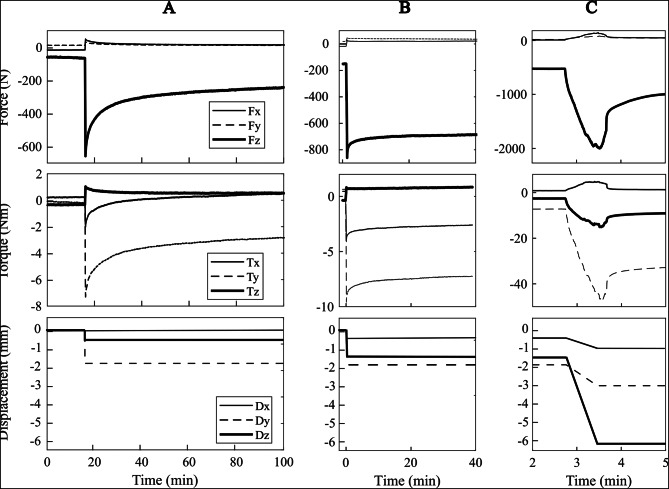
The force (top row) and torque (middle row) profiles recorded for the intact (**A**) and the implanted (**B**) humerus, under non-destructive loading. In (**C**), the profiles obtained by further incrementing the compression of the implanted specimen up to fracture (**C**). The displacement curve (bottom row) is obtained by connecting the displacement of the potting cup measured from the micro-CT images at each load step, synchronized with the start and end times of the force and displacement increase

The initial compression force for the intact humerus was equal to 67 N, while transversal force and torque components were below 15 N and 0.4 Nm. The physiological loading step was achieved by applying a 0.68 mm vertical displacement to the aluminum cup. The compression force reached 658 N (i.e., 594 N increment from the initial compression force), and the lateral displacement of the cup reached 1.80 mm. The stiffness of the intact humerus was 874 N/mm at the time of peak force. Compression decreased (relaxed) to 291 N (i.e., 55% of the peak compressive force) after 30 min. The initial compression force (pre-load) for the implanted humerus was equal to 150 N, with torque reaching 7 Nm in the frontal plane. The other force and torque components were comparable to those measured for the intact humerus. Applying 1.53 mm vertical displacement to the aluminum cup led the compression force to reach 861 N (i.e., 711 N increment from the initial compression force), as the cup moved medially by 1.89 mm. The vertical stiffness of the implanted humerus was 464 N/mm at peak force. Compression decreased (relaxed) to 690 N (i.e., 20% of the peak compressive force) after 30 min. As the vertical displacement increased to 6.12 mm, the compression force peaked at 2000 N and suddenly dropped to 1100 N. The compression force reached 1000 N in the following minute and remained substantially stable (Fig. 5).

**Fig. 6:**
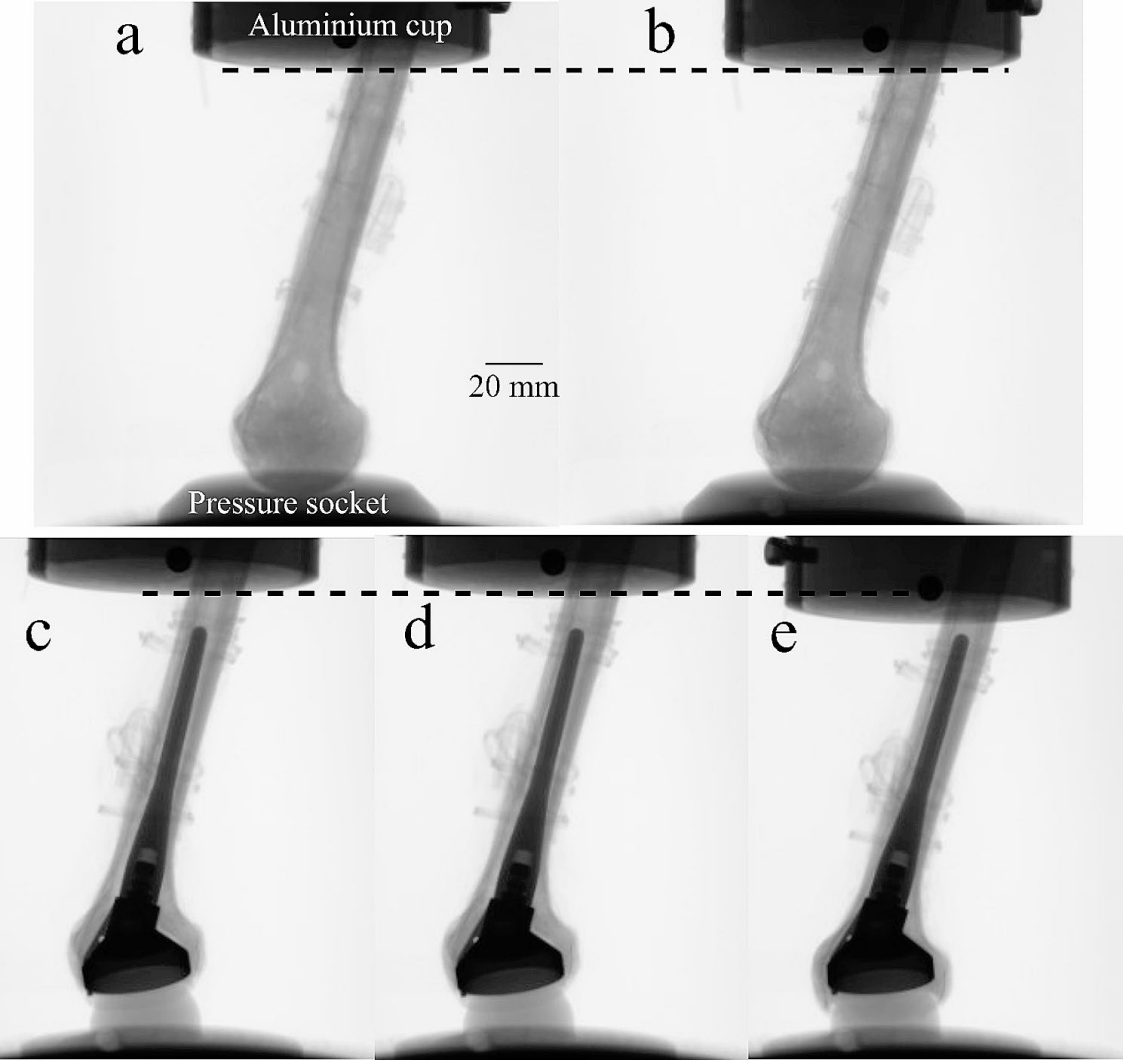
Micro-CT radiographic projections of the mechanical loading sequence, intact (top row) and once implanted (bottom row). Top row: (**a**) intact specimen in the initial position (pre-load) and (**b**) after application of a vertical compression inducing a physiological load increment. Bottom row: (**c**) implanted specimen in the initial position, (**d**) under a physiological load increment, and **d**) under a displacement causing a sudden drop of the compression force. The dashed line indicates the position of the aluminum potting cup in the initial position (pre-load)

The images displayed the intact and the implanted specimen during pre-load, physiological loading, and post-fracture of the implanted bone (Fig. 6).

**Fig. 7:**
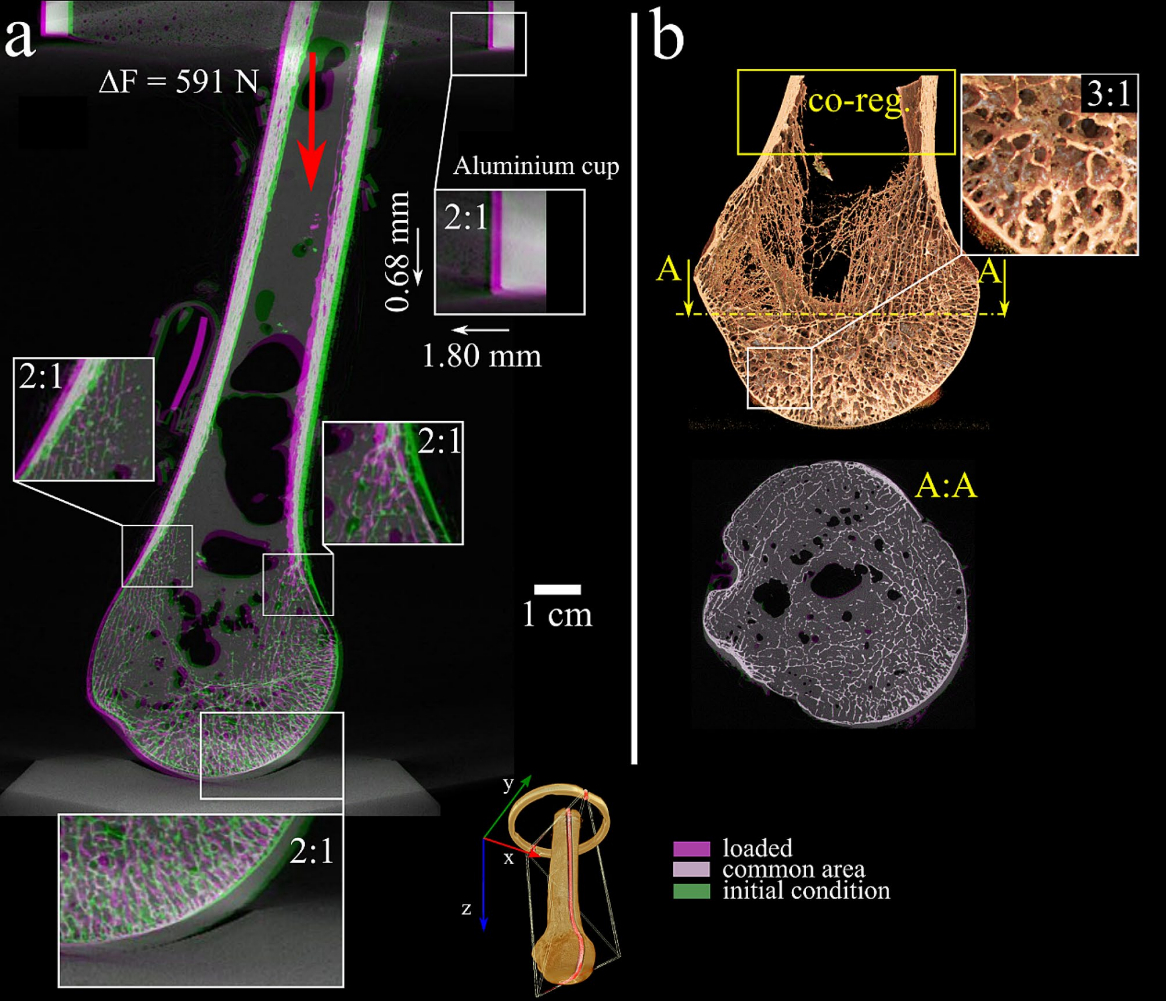
The micro-CT images of the intact humerus in the initial condition and after application of 591 N load increment, DF, displaying areas of overlap (grey), present in the initial condition only (green) and under application of the load increment (purple). The displacement of the entire specimen and the force exerted by the aluminum cup on the specimen (red arrow) is displayed on the left. On the right, the images co-registered at the height on the metaphysis displayed (quasi-frontal 3D representation) and a transversal cross-section (A: A) showing no appreciable movements in the entire bone microstructure

The intact humerus showed minimal movement at the contact between the articular surface and the pressure socket during the loading sequence, as its head and diaphysis rotated, moving laterally. The images co-registered at the proximal metaphysis showed no appreciable microstructural displacement over the entire head volume, indicating that most of the observed displacement was attributable to the bending of the diaphysis (Fig. 7).

**Fig. 8:**
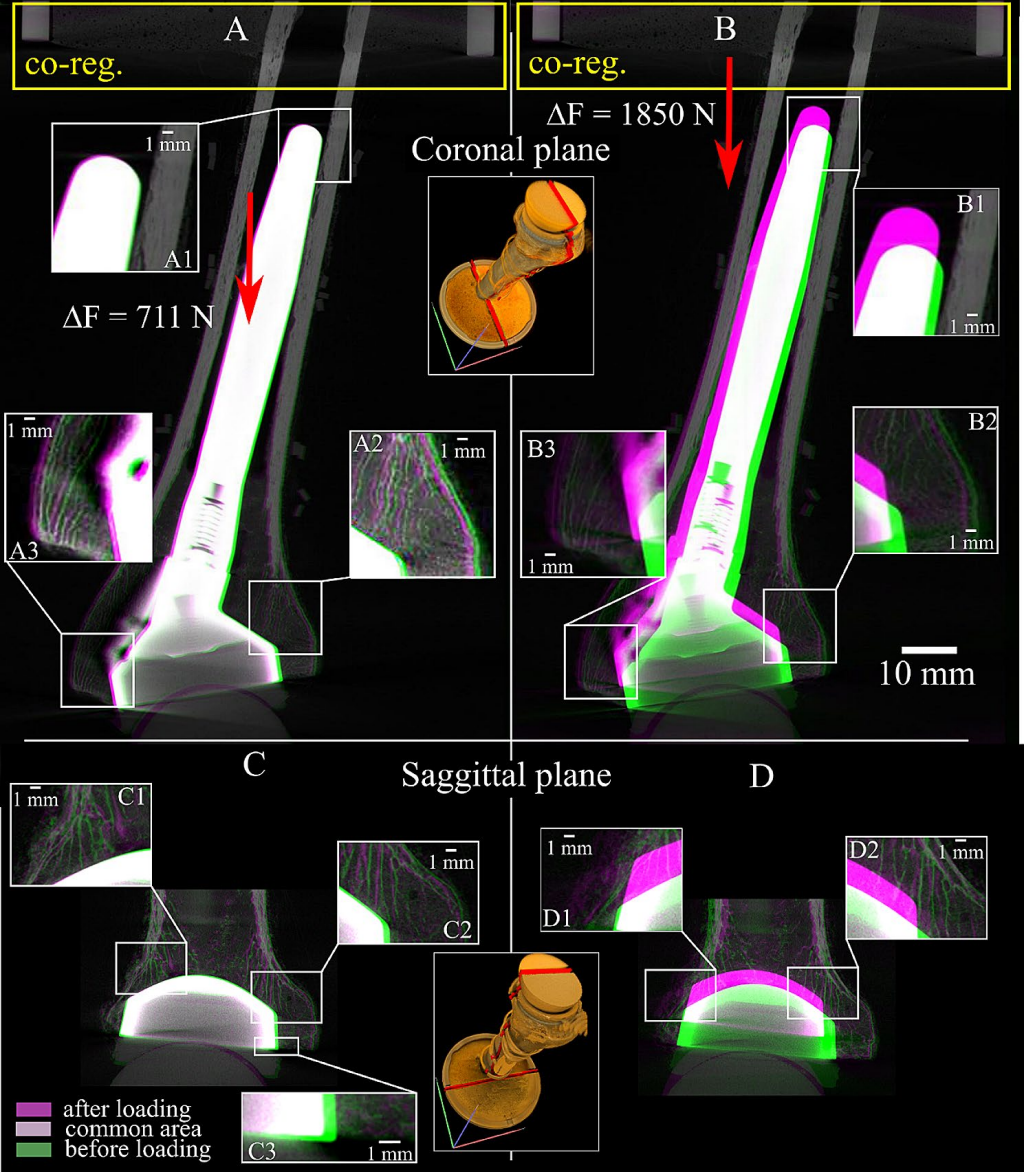
The micro-CT longitudinal cross-section images of the implanted humerus under non-destructive physiological load (**A**, **C**) and after failure (**B**, **D**). The images are co-registered to those in the initial condition, using the distal part of the specimen as a reference (yellow box). The compressive force exerted by the aluminum cup on the specimen (red arrow). Details of implant and trabeculae movements are also displayed along a sagittal and coronal section

**Fig. 9:**
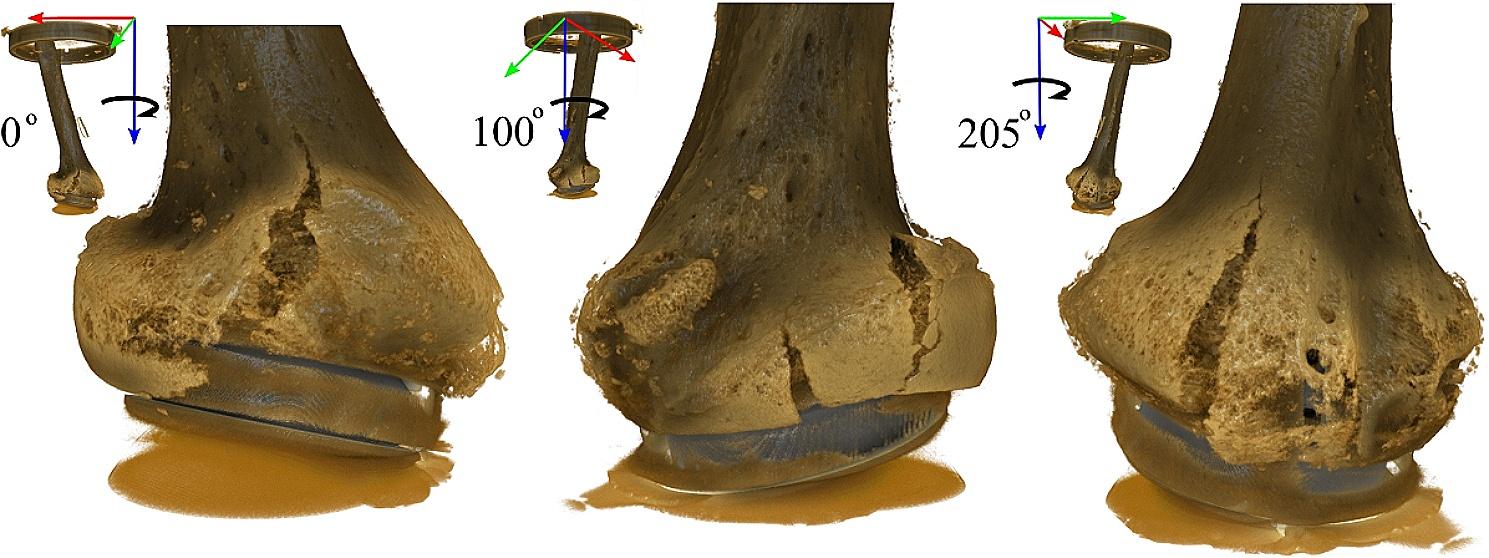
Rendering (in brown color) of the three longitudinal cracks opened from the rim of the implant cavity, visible from three different perspectives described in the top-right corner of each image

The implanted specimen rotated about the distal tip of the implant, where minimal displacement was observed, as the proximal specimen moved laterally (Fig. 8A). The displacement of single trabeculae in contact with the stem (made of titanium) was clearly visible in the coronal section (Fig. 8, C), where a 0.180–0.270 mm axial translation of the implant with respect to the rim could be measured (Fig. 8, C3). However, similar observations were difficult in the sagittal plane, showing metal artifacts in some areas close to the metaphyseal component (made of CoCr). As the specimen failed, the implant displaced distally and laterally with respect to the distal specimen, crushing the supporting trabecular bone (Fig. 8, D2) and breaking through the cortex (Fig. 8, D1). Three major longitudinal cracks opened in the bone from the rim of the implant cavity as the metaphyseal circumference increased, allowing the implant to migrate distally (Fig. 9).

## Discussion

The present study aimed at developing a novel protocol for studying the biomechanical failure of the humeral component of a reverse shoulder replacement. The protocol successfully provided the first available images of the entire microstructure of the proximal humerus under fully controlled loading and after fracture. The specimen’s stiffness approximately halved after implantation, yet provided stability to the implant under an extreme physiological loading, selected to maximize the axial migration of the implant. The images of the entire implant volume also allowed inferring the failure mechanism, by which the distal implant migration caused a combination of compression and circumferential expansion to the bone, which may explain the longitudinal cracks opening of the cortex from the cortical rim of the implant resection line. Ultimately, the present results demonstrate a novel use of large-volume micro-CT scanning and concomitant mechanical testing to analyze the biomechanical failure of the humeral component of reverse shoulder arthroplasty.

A comprehensive risk analysis of biomechanical failure benefits from information over the entire implant volume of displacements under load, relative to intact conditions. The present analysis showed similar stiffness of the intact and the implanted specimen in the transversal plane (Fig. 5), while the axial stiffness nearly halved after implantation. The 0.18–0.27 mm bone-implant movement measured at the rim of the host cavity (Fig. 8, C3) is consistent with that measured earlier in a stemless humeral implant (0.125 ± 0.064 mm) using optical methods [24], which later was found to be below the safe threshold for fibrotic tissue differentiation over the entire interface surface, as calculated using finite-element modeling [25]. Another interesting finding of the present analysis resides in the reduced force relaxation capacity of the bone after implantation, providing another poorly investigated effect of implantation on the thermomechanical properties of the natural humerus likely to have relevance for implant stability. Regarding failure, the micro-CT images enable inferring the failure mechanism, by which the increase of bone circumference as the implant moved distally revealed a combination of compression and circumferential tension, explaining the longitudinal cracks of the tuberosity opening from the rim of the implant cavity. Such fracture type is consistent with peri-prosthetic fracture classification by Campbell et al. (1998) [26]. Therefore, the present analysis demonstrates the utility of the testing protocol, although the single measurement does not allow a complete stability analysis of implant design and surgical procedure.

The research context for the present study consists of previous large-volume microstructural imaging studies of different anatomical regions [6–8, 20] and in vitro stability studies of the implanted humerus using different experimental methodologies [24, 27]. The displacement of the bone microstructure over the entire intact specimen was obtained similarly to earlier imaging of the femur and the spine [4, 6, 19]. A metal image artifact caused by the CoCr metaphyseal component (36 mm external diameter, 3–9 mm wall thickness) of the implant (Aequalis reversed II stem) was mostly localized in the proximal-lateral side of the specimen (where the implant has also a protruding anti-rotational feature), while displaying single trabeculae up to the interface with the implant in other regions (Fig. 8). This is in line with a previous analysis of bone fragment migration in images of repaired tibial plateau fractures, presenting metal artifacts caused by the CoCr locking plate and screws [8]. Previous in vitro analyses of a humeral implant include reports of bone-implant movements at the rim of the host cavity for a collarless and stemless design (Sidus Stem-Free, Zimmer GmbH) under 820 N applied at 30º from the implant axis in the frontal plane [25], and for a collared stem design (Neer II, Wright Medical Technologies) under 200 N applied axially. The present results (0.180–0.270 mm) are higher than the average movement of both the stemless design (0.125 ± 0.064 mm) and the collared stem design (< 0.04 mm) by an extent that differences in load magnitude and direction can explain. Therefore, the present protocol complements and expands current technologies for studying the biomechanical failure of humeral implants.

The major limitation of the present study resides in some metal (CoCr) artifacts surrounding the metaphyseal component, which may complicate further analyses in parts of that region. Using less dense, such as titanium- (as already done for this implant’s stem) or even aluminum-based alloys for it would substantially reduce these and allow to accurately calculate peri-prosthetic bone deformation via Digital Volume Correlation [6, 7, 20, 29]. Nevertheless, the available information can inform finite-element methods for determining peri-prosthetic bone strain and the relative motion over the entire bone-implant interface [25]. Another limitation resides in the quasi-static nature of the load applied, as lower damage tolerance and post-yield deformation likely occur at higher loading rates [28].

The present protocol provides a quasi-static response of the implant, which is consistent and directly comparable to earlier optical and planar radiographic studies [24, 27]. Finally, the loading direction used in the present study caused a tuberosity fracture by maximizing, among possible glenohumeral force directions, the alignment between the glenohumeral contact force and the longitudinal axis of the humerus. Different loading directions may cause more distal fractures of the implant by increasing the bending of the diaphysis over that used here. The load direction analyzed here provides a safe estimate of implant stability under a worst-case scenario of normal daily activity.

## Conclusion

This experimental protocol shows the integration of large-volume micro-CT scanning alongside concurrent mechanical testing, to elucidate the micro-mechanics of the humeral component in reverse shoulder arthroplasty under load. This also provides a framework for evaluating the risk associated with anticipated biomechanical failure mechanisms commonly linked to revision surgeries, due to biomechanical complications in reverse shoulder implants.

## Data Availability

The datasets used and analysed during the current study are available from the corresponding author on reasonable request.
